# Action Mechanisms of Du-Huo-Ji-Sheng-Tang on Cartilage Degradation in a Rabbit Model of Osteoarthritis

**DOI:** 10.1093/ecam/neq002

**Published:** 2011-03-20

**Authors:** Chao-Wei Chen, Jian Sun, Yu-Mei Li, Pi-An Shen, Yong-Qiang Chen

**Affiliations:** Department of Orthopaedics and Traumatology, Shanghai Municipal Hospital of Traditional Chinese Medicine, Shanghai 200071, China

## Abstract

Du-Huo-Ji-Sheng-Tang (DHJST) is a traditional Chinese herbal medicine used to treat osteoarthritis. In the present study, the therapeutic effect of DHJST on cartilage degradation in a rabbit model of osteoarthritis was investigated. In the knee joints of rabbits, anterior cruciate ligament transection (ACLT) was performed to induce experimental osteoarthritis. At the end of the sixth week, 30 rabbits with ACLT were divided into six groups, control group, DHJST group and Osaminethacine (OSA) group, which were followed for another 4 weeks. The other three groups of rabbits with ACLT were untreated with DHJST or OSA, which were sacrificed after 6 weeks, and served as 6-week time point controls. Results indicated that at the end of the sixth week after surgery, there was a significantly histological degeneration in the control group compared with the normal group. In the control group, the mean score for histological degeneration were further increases at 10th week, and there was a significantly lower mean score for histological degeneration in the DHJST group compared with the control group. To research the potential mechanism, the expression level of VEGF and HIF-1**α** were detected. The expression of VEGF mRNA and HIF-1**α** mRNA are low in normal group, while the activities increase gradually in the control group. However, compared to that of the same time point model group, activity of VEGF and HIF-1**α** decreased significantly in DHJST group. In conclusion, DHJST exerts significant therapeutic effect on osteoarthritis rabbits, and mechanisms are associated with inhibition of VEGF and HIF-1**α** expression.

## 1. Introduction

Osteoarthritis (OA) is a progressive and debilitating disease that may take years to clinically manifest in affected individuals. Cartilage aging and chondrocyte senescence play an important role in the pathogenesis and development of OA. During the development of OA, there is often a focal increase in cell death. Lots of reports revealed that chondrocyte senescence contributes to the risk of cartilage degeneration by decreasing the ability of chondrocytes to maintain and repair the articular cartilage tissue [1, 2]. Since it is theorized that each chondrocyte is responsible for the maintenance of the extracellular matrix surrounding it, and that matrix molecules produced in one region of the tissue have a very limited ability to traverse the tissue, the focal loss of viable cells could be partially responsible for the tissues inability to repair minor damage [3].

Currently, there is not a cure for OA, and available treatments only slow the progression of disease [4–6]. For the last 20 years, Traditional Chinese Medicine (TCM) has seen significant advancement against OA, such as in improving patients' clinical findings, inhibiting inflammatory reaction and cartilage degeneration [7, 8]. *In vivo* and *in vitro* study also showed that Chinese herbs formula is of multiple comprehensive actions against OA [9–12]. Du-Huo-Ji-Sheng-Tang, which is composed of *Radix Angelicae Pubescentis, Herba Taxilli, Herba Taxilli, Radix Acanthopanacis Bidentatae, Herba Asari, Radix Gentianae Macrophyllae, Cortex Cinnamomi* and *Poria*, has been widely used for treating OA. It can improve clinical symptoms, knee function and quality of life for patients [13]. Based on our knowledge about OA pathogenesis in TCM, we investigated the effect of DHJST on preventing cartilage degeneration of OA in rabbits and observed its mechanisms.

## 2. Methods

### 2.1. Materials

The Dead End colorimetric apoptosis detection system (TUNEL assay) was obtained from Nanjing Jiancheng Biotech Company (China). Trizol total RNA extraction kit, DNA molecular marker, SYBR Premix EX TaqTM and Perfect Real Time kit were purchased from the Invitrogen Co. (USA). Herbs in DJST (composed of *Radix Angelicae Pubescentis, Herba Taxilli, Herba Taxilli, Radix Acanthopanacis Bidentatae, Herba Asari, Radix Gentianae Macrophyllae, Cortex Cinnamomi* and *Poria*, provided by Shanghai Huayu Chinese Herbs Co. Ltd., China) were accredited by a pharmacognosist according to standard protocols, prepared by Shanghai Municipal Hospital of Traditional Chinese Medicine, the components were mixed in order with the ratio of 2 : 1 : 1 : 1 : 0.2 : 1 : 0.3 : 1 (dry weight), The drugs were extracted with standard methods according to Chinese Pharmacopoeia (China Pharmacopoeia and Committee, 2000). These crude drugs were soaked in distilled water and boiled for 30 min twice, and the drug solution was filtered through a mesh, then the filtrate was concentrated to 4 g mL^−1^ by a vacuum pump and then stored at –20°C until use. Osaminethacine (OSA), each tablet contains 25 mg indomethacin and 75 mg aminoglucose (supplied by Hebei Jizhong Pharmaceutical Co. Ltd., China), was used as positive control medicine.

### 2.2. Rabbits and Tissue Preparation

Forty-eight-month-old New Zealand white rabbits were supplied by the Experimental Animal Centre to Chinese Academy of Science. Thirty rabbits underwent unilateral anterior cruciate ligament transection (ACLT) under general anesthesia. The normal rabbits did not undergo a procedure.

At the end of the sixth week, the 30 rabbits with ACLT were divided into six groups, control group, DHJST group and OSA group, which were followed for another 4 weeks. The other three groups of rabbits with ACLT were untreated with DHJST or OSA, which were sacrificed after 6 weeks, and served as 6-week time point controls. The decoctions were orally administrated at dosage 0.923 g (kg^−1^·d^−1^) for 4 weeks, rabbits in the OSA group were administered OSA 0.09 g (kg^−1^·d^−1^) for a period of 4 weeks, model control group and normal group were orally administrated with same volume of physiological saline.

Rabbits were sacrificed for sampling. The protocol received approval of the committee for Animal Experiments of the University Medical Centre, Shanghai, China. Rabbit tibiae was removed and tibia articular cartilage was immediately stored at –80°C. The tibiae were carefully dissected and cleared from adjacent muscle and immediately fixed in 10% formalin for 24 hours. Tibiae were subsequently decalcified for 4 weeks in 100 g/L ethylenediamine tetraacetic acid, washed in PBS, dehydrated through a series of ethanol and embedded in paraffin in a standardized way to ensure proper orientation. Paraffin tissue sections (7 *μ*m) were cut in a standardized way. Sections were deparaffinized for histochemical analyses.

### 2.3. Histological Assessment of Cartilage

Histological assessment was performed on the femorotibial joints of rabbits in the DHJST group, OSA group and control groups. The sections were stained with Safranin O-fast green. These histological sections from each site were evaluated using a modified Mankin grading system [14] (Table 1) by two independent, board-certified veterinary pathologists.

### 2.4. TUNEL Assay

H_2_O_2_-induced apoptosis was detected by performing the terminal deoxynucleotidedyl transferase-mediated dUTP nick end-labeling (TUNEL) assay using an Apo-DirectTM Kit (CalBiochem, San Diego, CA). TUNEL was performed according to the manufacturer's instructions. Briefly, after pretreatments and exposure to H_2_O_2_, cells were harvested, washed, fixed, permeabilized and labeled for DNA strand breaks, then analyzed on a Coulter Epics Elite flow cytometer (Beckman-Coulter, Miami, USA). All assays were carried out in triplicate.

### 2.5. Fluorescent Quantitative PCR

Total RNA was extracted from 50 mg of cartilage in tibiae articular using Trizol reagent. Synthesis of cDNA was performed using 4 *μ*g of total RNA per sample with random primers and reagents contained in the Revert Aid First Strand cDNA synthesis kit. Two micro liters (2 *μ*L) of each sample was used for real-time PCR in a Rotor-Gene 2000 system (Australia). Relative quantitation was calculated using delta cycle threshold (C_T_) relative quantitation. The threshold cycle (C_T_) for the endogenous control CAPDH mRNA and the target signals were determined, and the relative RNA quantification was calculated using comparative 2^−ΔΔCt^ method [15] where ΔΔCt = (C_T,Target _ − C_T,GAPDH _)_responder_ − (C_T,Target _ − C_T,GAPDH _)_not-responder_

Sequences of primers used for vascular endothelial growth factor (VEGF) are: 5′-TGCCCACCGAGGAGTTCA-3(forward) and 5′-GGCCCTGGTGAGGTTTGAT-3(reverse) (product length: 75 bp).
Sequences of primers used for hypoxia-inducible factor-1*α* (HIF-1*α*) are: 5′-CAGTGCAAAAGACAGGTGGAAG-3(forward) and 5′-CCCTGTATGGTGGTGATGTTGT-3(reverse) (product length: 107 bp).
Sequences of primers used for GAPDH are: 5′-CCGAGGGCCCACTAAAGG-3(forward) and 5′-GCTGTTGAAGTCACAGGAGACAA-3(reverse) (product length: 88 bp).

### 2.6. Statistical Analysis

All results were expressed as mean and standard deviation (SD). Measurement data were analyzed using a one-way analysis of variances (ANOVA, SPSS 11.0). Rank data were analyzed with ridit. The result of *P* < .05 was considered to be statistically significant.

## 3. Results

### 3.1. ACLT-Induced Histological Degeneration in Rabbits Were Reduced by DHJST

**Figure 1:**
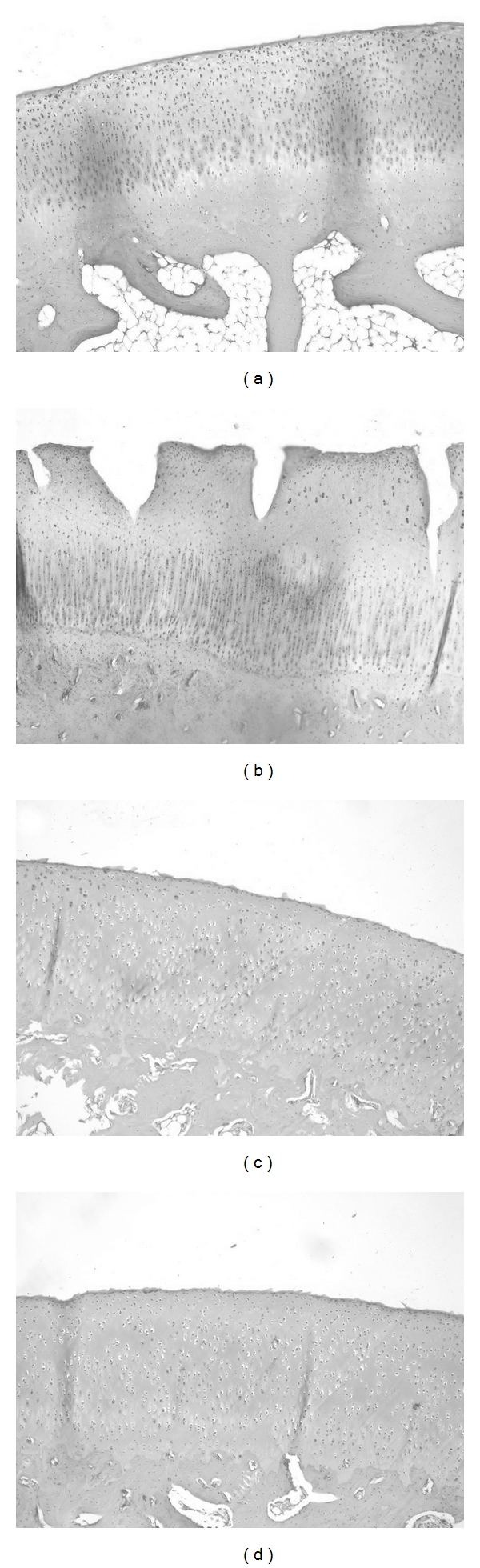
Histological examination of cartilage (Safranin O-fast green staining, ×100).

**Figure 2:**
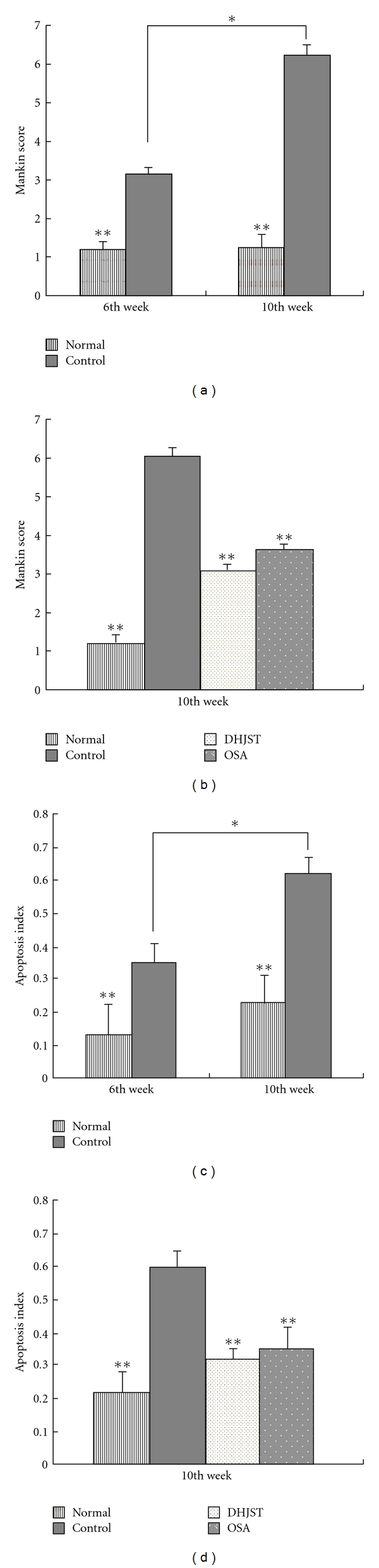
The influence of DHJST on histological parameters and apoptotic of chondrocytes. Results were presented as means ± SD (*n* = 5 per group) in each column. *P < .01 versus the osteoarthritis control group at the 6th week, **P < .01, compared with those in the osteoarthritis control group.

Representative histological sections are shown in Figure 1. At the end of 10th week, we observed a normal integrity of the articular surface in normal group (Figure 1(a)). In control group, with increased degeneration, there was a loss of Safranin O-fast green staining accompanying cleft formation that extended into the middle and deep zones (Figure 1(b)), and in DHJST group, there was lost with early mild fibrillation, and a loss of Safranin O-fast green staining was observed along with chondrocyte clustering (Figure 1(c)). OSA group, with loss of Safranin O-fast green staining these changes were similar to DHJST group (Figure 1(d)). At the end of the sixth week after surgery, there was a significantly histological differences in the control group compared with the normal group (*P* < .01). In the control group, the mean score for Safranin O-fast green staining were further increases at 10th week in comparison with that of at the 6th week (*P* < .01) (Figure 2(a)). At 10th weekend, there were significant differences in Mankin score between control group and DHJST group, there was a significantly lower mean score for Safranin O-fast green staining in the DHJST group compared with the control group (*P* < .01) (Figure 2(b)), and no statistically significant differences in the Mankin score between DHJST group and OSA group (*P* >.05).

### 3.2. DHJST Inhibited Cartilage Cells Apoptosis

At the end of the sixth week after surgery, the index of apoptosis cell in the control group compared with that in the normal group were significantly differences (*P* < .01). In the control group, the index of apoptosis cell was still greatly increased (from mean ± SD 0.35 ± 0.13 at the sixth week to 0.62 ± 0.10 at the 10th week; *P* < .01) (Figure 2(c)). At the end of 10th week, compared with DHJST group, the index of apoptosis cell increases remarkably in the control group (*P* < .01) (Figure 2(d)). There were no significant differences in apoptosis cell index between OSA group and DHJST group (*P* >.05).

### 3.3. Inhibition of VEGF and HIF-1*α* Expression in Cartilage Cells Are Associated with DHJST Exerts Therapeutic Effect

Fluorescent quantitative PCR indicated that the expression of VEGF mRNA and HIF-1*α* mRNA are low in normal group; while the activities increase gradually and significantly in the control group in comparison with that of normal group (*P* < .01); In control group, HIF-1*α* in articular chondrocytes begins to increase at the sixth week after surgery, further increases at 10th week in comparison with that of at the sixth week (mean ± SD 94.21 ± 20.12 at the sixth week, and 160.35 ± 72.27 at the 10th week; *P * < .05) (Table 2). At 10th weekend, The expression of VEGF mRNA and HIF-1*α* mRNA in DHJST group was significantly decreased as compared with that in control group after administration (*P* < .01). Although the VEGF mRNA and HIF-1*α* mRNA level in OSA group tended to be reduced by 4 weeks of OSA treatment as compared with that in control group, there was no significant difference (Table 3).

## 4. Discussion

The ACLT model is one of the most widely used experimental models of OA. Rabbit ACLT is increasingly being used in OA studies [16] because disease onset is rapid. The use of experimental ACLT models is of particular clinical relevance, since rupture of the ACL occurs in humans and also leads to the development of OA. Study indicates that large amount of chondrocytes apoptosis exists during progressive stage of OA in rabbits induced by surgery. Chondrocyte apoptosis is one of the characteristic changes of OA cartilage. Pathological apoptosis under the condition of harmful stimulation could result in the release of chemotaxis or inflammatory factors, which in turn aggravates cartilage injury and activates pain [17]. Therefore, chondrocyte apoptosis inhibiting might be an important strategy against degenerative changes in rabbits knee joints. Chondrocytes apoptosis index in DHJST group significantly decreases in comparison with that of in the model group, suggesting that inhibition of chondrocyte apoptosis might be one of the important mechanisms of DHJST against degenerative changes.

Previous studies have shown the hypoxic nature of the articular chondrocytes' microenvironment. Interestingly, osteoarthritic joints displayed further decreased oxygen levels, role of hypoxia during the pathogenesis of OA [18]. A recent study described that in articular chondrocytes catabolic and hypoxic stress are strong inducers of the transcription factor HIF-1, which is the principal regulator of cellular adaptation to low oxygen levels. HIF-1 is of pivotal importance for survival and growth arrest of chondrocytes during cartilage development as well as energy generation and matrix synthesis of chondrocytes in healthy as well as osteoarthritic cartilage. In osteoarthritic cartilage, the protein levels of HIF-1 are significantly increased and its activity correlates to the severity of degenerative cartilage changes [19–24]. The present work documents the importance of the transcription factor HIF-1*α* for maintaining the integrity of hypoxic articular cartilage. The surplus levels of HIF-1*α* coincided with increased apoptosis of articular chondrocytes and led to degenerative changes in rabbits knee joints. After administration of DHJST, HIF-1*α* expression decreases, in comparison with that of model group. The results show that action of DHJST on regulating function of chondrocytes, might through inhibiting activity of HIF-1*α*.

It is interesting that the different of the expression of VEGF and HIF-1*α* between DHJST group and OSA group is significant after administration. Several studies have demonstrated that certain nonsteroidal anti-inflammatory drugs, such as indomethacin, cause anti-inflammatory effects independent of HIF-1*α* activity, because indomethacin failed to inhibit phosphatidylinositol 3-kinase or extracellular signal-regulated kinase/mitogen-activated protein kinase pathway, as well as Erk activity [25]. In contrast, indomethacin is able to activate peroxisome proliferator-activated receptor-*γ*, which is not sensitive to sodium salicylate or aspirin. Inhibition of phosphatidylinositol 3-kinase or Erk pathway is related to inhibition of HIF-1*α* and angiogenic factors [26].

**Figure 3:**
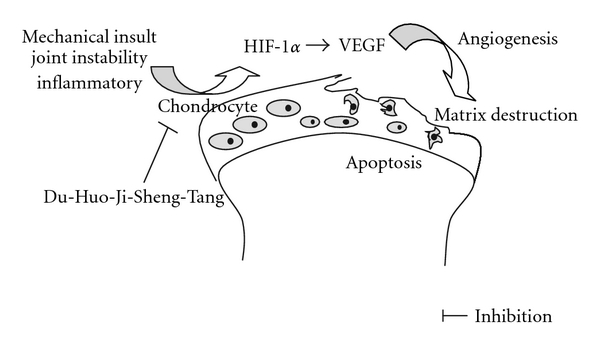
Hypothetical protective mechanisms of DHJST on cartilage degradation.

Hypoxia and various growth factors/cytokines enhance VEGF expression [27–30]. VEGF is not only one of the most important angiogenesis factors, but is involved also in inflammatory processes in OA and contribute to symptoms such as pain and swelling by targeting the synovial membranes [31–34], chronic inflammatory responses are often associated with the production of angiogenic factors that induce vascular proliferation, and in rheumatoid arthritis VEGF is highly expressed in synovial cells [35–40]. Since mechanical overload is one of the causative factors for OA, we studied its effect on VEGF expression on rabbit's cartilage that the expression of VEGF decreases after administration of DHJST, the result indicates that DHJST can protect the articular cartilage through suppressing the VEGF expression in chondrocytes (Figure 3).

## 5. Conclusions

Up to now, the bioactive ingredients in DHJST is unknown, but our study indicates that DHJST exerts significant therapeutic effect on OA in rabbits, through mechanisms of inhibiting chondrocytes apoptosis, and regulating the expression of VEGF, HIF-1*α* in chondrocytes. This provides scientific evidence for clinic to apply DHJST in treating OA.

## Funding

The Natural Science Foundation of Shanghai (grant number: 08JC1418800).

## Figures and Tables

**Table 1:** Criteria (grading) for histologic evaluation.

Score	Structure	Chondrocyte loss	Safranin O-fast green staining	Tidemark integrity
0	Normal	No decrease in cells	Uniform staining throughout articular cartilage	Intact
1	Surface irregularities	minimal Decrease in cells	Loss of staining in the superficial zone for less than one-half of the length of the condyle or plateau	Crossed by blood vessels
2	>3 superficial clefts	Moderate decrease in cells	Loss of staining in the superficial zone for one-half or more of the length of the condyle or plateau	
3	1–3 superficial clefts	Marked decrease in cells	Loss of staining in the superficial and middle zones for less than one-half of the length of the condyle or plateau	
4	1–3 clefts extending into the middle zone	very extensive decrease in cells	Loss of staining in the superficial and middle zones for one-half or more of the length of the condyle or plateau	
5	1–3 clefts extending into the deep zone		Loss of staining in all three zones for less than one-half of the length of the condyle or plateau	
6	Clefts extending to calcified cartilage		Loss of staining in all three zones for one-half or more of the length of the condyle or plateau	

**Table 2:** General characteristics of HIF-1*α* mRNA and VEGF mRNA between 6 and 10 weeks.

Treatments	Sixth week		10th week	
	VEGF	HIF-1*α*	VEGF	HIF-1*α*
Normal group	0.55 ± 0.27**	0.71 ± 0.15**	0.55 ± 0.23**	0.69 ± 0.17**
Control group	80.01 ± 6.07	94.21 ± 20.12	125.01 ± 26.07*	160.35 ± 72.27*

Values as means ± SD were obtained from each group of 5 animals.

*P  < .05 compared with control group animals at the sixth week.

**P  < .01 compared with control group animals at the same time point control group.

**Table 3:** The influence of DHJST on HIF-1*α* mRNA and VEGF mRNA in the cartilage cells of rabbits at 10th week.

Treatments	*n*	10th week	
		VEGF	HIF-1*α*
Normal group	5	0.55 ± 0.23**	0.69 ± 0.17**
Control group	5	125.01 ± 26.07	160.35 ± 72.27
DHJST group	5	20.34 ± 2.16**	5.44 ± 2.36**
OSA group	5	113.42 ± 42.32^†^	120.44 ± 81.12^†^

Values as means ± SD.

^†^ *P * > .05 compared with control group animals.

**P < .01 compared with control group animals.
